# Minor Injuries in Children

**Published:** 1911-06-10

**Authors:** 


### DISEASES OF CHILDREN.

### MINOR INJURIES IN CHILDREN.

ACUTE or sub-acute inflammation of bone in children starts in the vascular newly-formed tissue that lies immediately under the epiphysis. Through this tissue occurs the lesion in a separation of an epiphysis. This anatomical fact suggests the possibility of some connection between the two. The suggestion takes more definite shape when two other facts are remembered. First, that a large proportion of cases of primary inflammation of bone give a history of some injury, thought at the time to be trivial, before the onset of the disease. Secondly, that some cases of separated epiphysis suppurate without ever being compound—that is, a primary inflammation of the bone occurs in them.

An epiphysis may be separated from its diaphysis without there being any displacement. So firm is the attachment of the periosteum to the cartilage of the epiphysis that it is never torn away. Hence the periosteum goes with the epiphysis, and displacement can only occur if the periosteum be ruptured higher up and the diaphysis herniate out through it. That such separations occur without displacement has been proved by dissection and by the *x*-rays. But the injury may not be even a complete separation, there may be only a partial separation or fissure fracture going across the bone under the epiphysis.

Such injuries as these cannot be diagnosed without the *x*-rays; they give rise to no objective symptoms beyond some tenderness in the region of the epiphysis, which may also come from a stretched or partially-ruptured ligament or a bruise of the subcutaneous tissues.

The anatomical and pathological facts detailed above have an important practical bearing. First, any small injury in the limb of a child or youth may mean some lesion of the bone; many cases diagnosed as sprain, ruptured ligaments, or bruises on the bone belong to this class. Secondly, every injury to the bone in such a patient is a potential case of acute osteo-myelitis or tuberculous disease; and, therefore, every injury occurring near the end of a long bone in an individual whose epiphyses have not joined may result in one of these.

The results of acute bone disease are so far-reaching that it is good treatment to keep a hundred children in bed or away from school for three or four days to a week, if by so doing one can be saved from death, from a severe illness, or the loss of a limb or the function of that limb.

In other words, all such minor injuries should be treated by rest in bed until all pain and tenderness has gone and been gone for two days. If the child have much animal spirits in him it will be necessary to put the limb up in a splint to prevent use of it.

There is one further point that needs emphasising. If the injury be associated with an abrasion of the skin, a way in has been opened to such organisms as are there. These abrasions, when associated with deep-seated tenderness, should be looked upon with the gravest suspicion, and care be taken to prevent suppuration in them, however slight.

